# Self-organisation and convection of confined magnetotactic bacteria

**DOI:** 10.1038/s41598-020-70270-0

**Published:** 2020-08-11

**Authors:** Albane Théry, Lucas Le Nagard, Jean-Christophe Ono-dit-Biot, Cécile Fradin, Kari Dalnoki-Veress, Eric Lauga

**Affiliations:** 1 Department of Applied Mathematics and Theoretical Physics, University of Cambridge, Cambridge, CB3 0WA UK; 2 Department of Physics and Astronomy, McMaster University, 1280 Main St. W, Hamilton, ON L8S 4M1 Canada; 3 UMR CNRS Gulliver 7083, ESPCI Paris, PSL Research University, 75005 Paris, France

**Keywords:** Biological physics, Fluid dynamics

## Abstract

Collective motion is found at all scales in biological and artificial systems, and extensive research is devoted to describing the interplay between interactions and external cues in collective dynamics. Magnetotactic bacteria constitute a remarkable example of living organisms for which motion can be easily controlled remotely. Here, we report a new type of collective motion where a uniform distribution of magnetotactic bacteria is rendered unstable by a magnetic field. A new state of “bacterial magneto-convection” results, wherein bacterial plumes emerge spontaneously perpendicular to an interface and develop into self-sustained flow convection cells. While there are similarities to gravity driven bioconvection and the Rayleigh–Bénard instability, these rely on a density mismatch between layers of the fluids. Remarkably, here no external forces are applied on the fluid and the magnetic field only exerts an external torque aligning magnetotactic bacteria with the field. Using a theoretical model based on hydrodynamic singularities, we capture quantitatively the instability and the observed long-time growth. Bacterial magneto-convection represents a new class of collective behaviour resulting only from the balance between hydrodynamic interactions and external alignment.

## Introduction

Examples of collective motion can be found in many biological systems over a wide range of length scales^1^, from animal herds^2^, bird flocks^3^ and schools of fish^4^, to individual cells^5^. Microorganisms swimming in viscous fluids at low Reynolds numbers can form coherent patterns that depend on the propulsion modes of the swimmers^6–8^. For instance, randomly-swimming cells such as bacteria^9^ and spermatozoa^10^ are able to self-organise into large-scale coherent structures.

In contrast with the random motion of many cells, micro-swimmers and artificial swimmers can also bias their movement in response to external cues. The bias can arise actively, as in the cases of chemotaxis^11^ and phototaxis^12^, or passively, for example gyrotactic swimmers^13^, resulting in emergent pattern-formation in dense systems—a characteristic of collective locomotion. A classic example in this case is bioconvection, an instability initiated by the accumulation at an interface of gyrotactic^14^ or phototactic^15^ swimmers that are denser than the surrounding fluid. The resulting density stratification is unstable^16^, which leads to the formation of downward falling plumes^17, 18^. Boundaries and confinement^19, 20^ also strongly affect the swimming of microorganisms and their hydrodynamic interactions^21^. Although back-flow generated by boundaries tends to screen swimmer-swimmer interactions, the emergence of wall-driven attractive interactions has been observed, leading to orbiting^22^ and large-scale clustering at a wall^23^.

Magnetotactic bacteria (MTB) are prokaryotic micro-swimmers found in abundance in freshwater and marine habitats^24^. These bacteria can synthesize magnetosomes, membrane-bounded, magnetic nanocrystals often assembled into chain-like structures within the cytoplasm^25^. Magnetosomes confer to each cell a permanent magnetic dipole moment, forcing the swimming bacterium to align with external magnetic fields. The orientation and direction of motion of MTB can thus be manipulated remotely using controlled magnetic fields^26–28^. MTB can be used as a source of magnetosomes, which have demonstrated a great potential for medical applications due to their unmatched chemical purity and magnetic properties^29^. Furthermore, MTB have even been proposed as potential agents for targeted drug delivery^30^. A thorough understanding of the individual and collective properties of MTB is thus needed to fully exploit their potential for practical applications. The wall-mediated behaviour of single and pairs of MTB has been explored^31^, and attractive hydrodynamic interactions lead to clustering near a wall^32^. Various patterns and instabilities can be induced by the control and orientation of large swimmer populations^33^, including pearling of focused MTB in a thin capillary^34, 35^ or vortex formation in droplets^36^.

Here we report on a new type of collective motion observed in a population of confined MTB induced by the application of a magnetic field perpendicular to the confining walls. We find that a uniform distribution of magnetotactic bacteria is observed to self-organise spontaneously into bacterial plumes perpendicular to the wall, resulting in self-sustained convection cells. The observed plumes are reminiscent of bioconvection patterns and the flow generated resembles Rayleigh–Bénard convection cells^37^. However, an important difference exists between the phenomenon reported here and these well-studied instabilities. Both bioconvection and the Rayleigh–Bénard instability rely on a density mismatch between layers of the fluids, and thus on net (gravitational) forces applied to the fluid. In both these cases, removing the external force from the governing equations results in the absence of convective cells. In contrast, for the phenomenon reported here, no external forces are applied on the fluid and the magnetic field only exerts an external torque aligning MTB with the field^34^. A different mechanism thus needs to be proposed to describe the plumes and flow patterns observed.

Using a theoretical model based on hydrodynamic singularities, we show that the plumes result solely from the balance between the ordered state of the swimmers and both cell-cell and cell-wall hydrodynamic interactions. An instance of biased active matter related to previous examples in biological (phototactic, gyrotactic and chemotactic cells) and artificial systems (active phoretic particles in shear flows near surfaces), the self-sustained state reported here, which we term bacterial magneto-convection, represents therefore a new class of collective behaviour resulting only from the balance between hydrodynamic interactions and external alignment.

## Results

### Bacteria self organisation and plume formation

**Figure 1:**
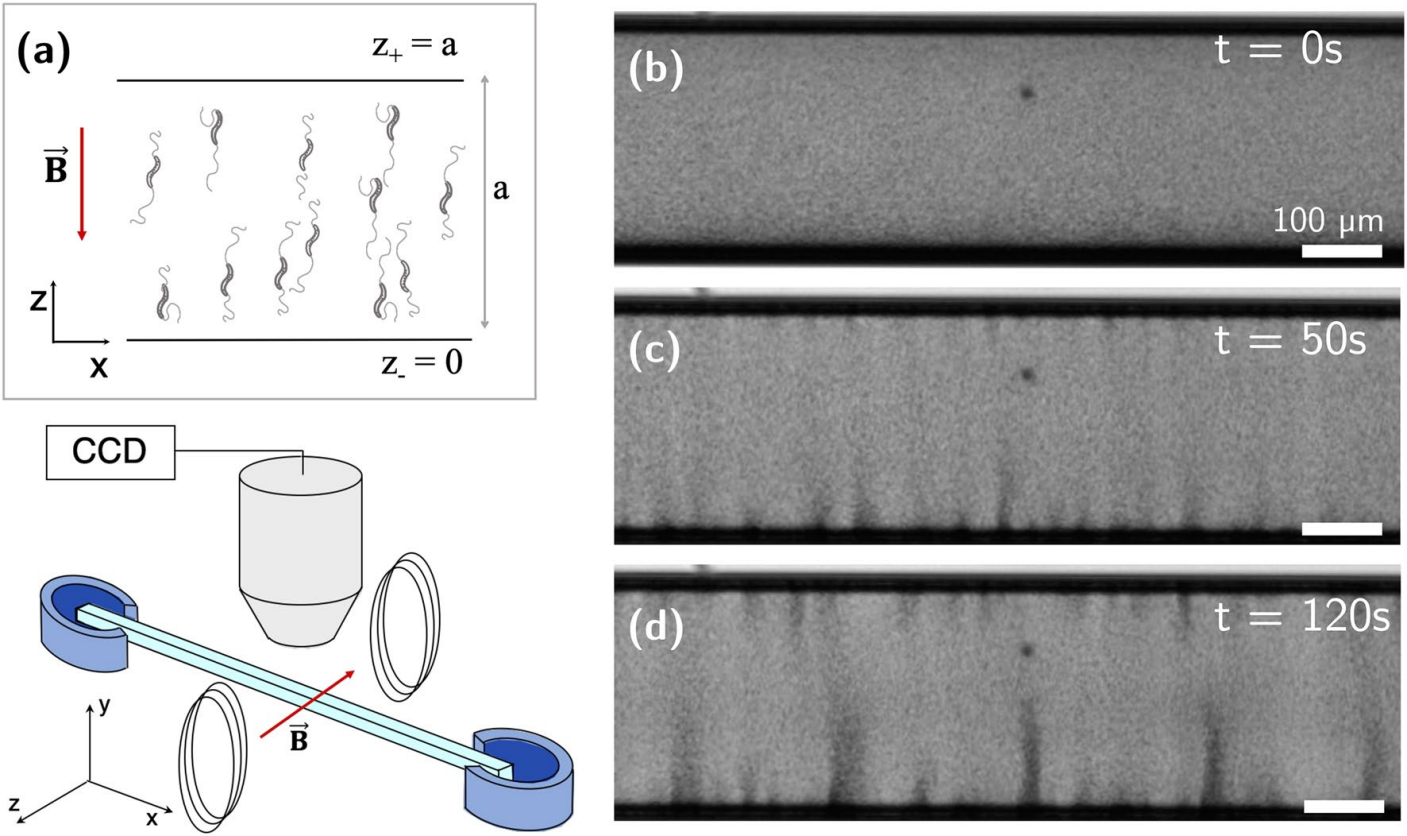
(**a**) Experimental set up and schematic view of the observed bacteria in the microscope under the magnetic field $\mathbf{B}$. A glass capillary with square cross-section and pore size ranging from $80~{\upmu \mathrm{m}}$ to $500~{\upmu \mathrm{m}}$, is held and sealed at both ends by two reservoirs filled with mineral oil. Two Helmholtz coils generate a uniform magnetic field and a microscope is used to image the bacteria. (**b**) Uniform distribution of bacteria in a capillary with dimensions $300~\upmu \mathrm{m}$ square at $t<0$. The magnetic field magnetic field is then turned on at $t=0$. (**c**) At $t=50~{\mathrm{s}}$, bacteria accumulate near the $z_-$ wall, and the concentration instability emerges. (**d**) At longer times, plumes grow and cluster until a steady flow is obtained at $t=2~\text {min}$.

In our experiments, bacteria of the strain *Magnetospirillum magneticum* AMB-1^38^ are confined to square capillary channels aligned along the *x*-axis. Capillaries are filled with a concentrated solution of MTB and an external magnetic field, **B**, is applied normal to the channel wall along the positive *z* direction (see schematic in Fig. 1a and Methods section). The field **B** is uniform throughout the capillary, with strength ranging from $1 \times 10^{-4}\,{\mathrm{T}}$ to $20 \times 10^{-4}\,{\mathrm{T}}$ (Fig. 1). The bacteria are confined within the tube by the walls $z_-$ and $z_+$ perpendicular to **B**, and by the walls $y_-$ and $y_+$ in the orthogonal directions. Images are acquired at $10~{\mathrm{fps}}$ and particle image velocimetry (PIV) is used to study the flow in the tubes, using bacteria as tracers. Some of the bacteria are non-motile and we observe the fluid flow generated by the plumes in the capillary. Cultures of motile and magnetic MTB were obtained through a racetrack selection method^39^ which results in a bacteria population dominated by a preferred polarity and therefore in bacteria that predominantly swim in one direction. Despite the selection, there is always a small minority population present with the reverse polarity which swims in the opposite direction. Due to the small size of that minority population, the reverse swimmers are neglected in our analysis.

In the absence of magnetic field, the bacteria are homogeneously distributed in the channel (Fig. 1b) with bacteria swimming in random directions. When the magnetic field is switched on, swimmers orient along the magnetic field **B**, and most motile bacteria start swimming towards the $z_-$ wall (Fig. 1a) with velocities ranging from $\sim 4$ to $10\, {\upmu \mathrm{ms}^{-1}}$. As bacteria accumulate at the $z_-$ wall, small clusters form and start growing. The time before the instability becomes visible depends strongly on both concentration and population of the bacteria, and ranges from a few seconds to $50 \,{s}$. As the clusters grow, they start forming plumes which advect bacteria through a strong backward flow (Fig. 1c). The plumes interact with their nearest neighbours and attract each other, leading to merging and formation of larger plumes, with ultimate separations between plumes comparable to the channel size (Fig. 1d) (see Supplementary movies SM1, SM2 and SM3). In the larger $500~{\upmu \mathrm{m}}$ capillaries we observe by changing the focus of the microscope along the *y*-direction, that the plumes form in the central region of the channel, as opposed to close to the walls.

We observe the plumes and measure their dynamics in 26 experiments carried out in different capillary sizes and bacterial concentrations. In a few experiments, the plumes do not appear or do not grow. The mobility of bacteria and their sensitivity to the magnetic field are two key parameters that impact the dynamics of the instability and the plume formation. The dynamical properties of the instability are quantified by the characteristic time, $\tau _p$, to reach the steady state. Experimentally $\tau _p$ ranges from $170\text {s}$ to $600 \text {s}$. Although no critical bacterial concentration can be determined, we find that lower concentrations of bacteria lead to larger $\tau _p$, and to formation of smaller plumes that do not reach the same late-time wavelength as concentrated systems.

Below a critical magnetic field, which depends on the bacteria population and concentration and ranges from $2.6 \times 10^{-4} \text {T}$ to $10 \times 10^{-4} \text {T}$, no plumes are observed. Above this critical field, plumes grow and evolve with dynamics unaltered by field strength (see Supplementary Material). The existence of a critical magnetic field can be understood by the necessity to have the bacteria aligned within a narrow orientation distribution along the magnetic field direction. All our experiments are carried out with magnetic fields much larger than the observed critical fields ($2 \,{\mathrm{mT}}$) to rule out any influence of the field strength and ensure that the bacteria remain perfectly aligned with the field (see details in Supplementary Material). We also note that at this field strength, magnetic dipole-dipole interactions between neighbouring bacteria are negligible compared to those with the applied magnetic field.

**Figure 2:**
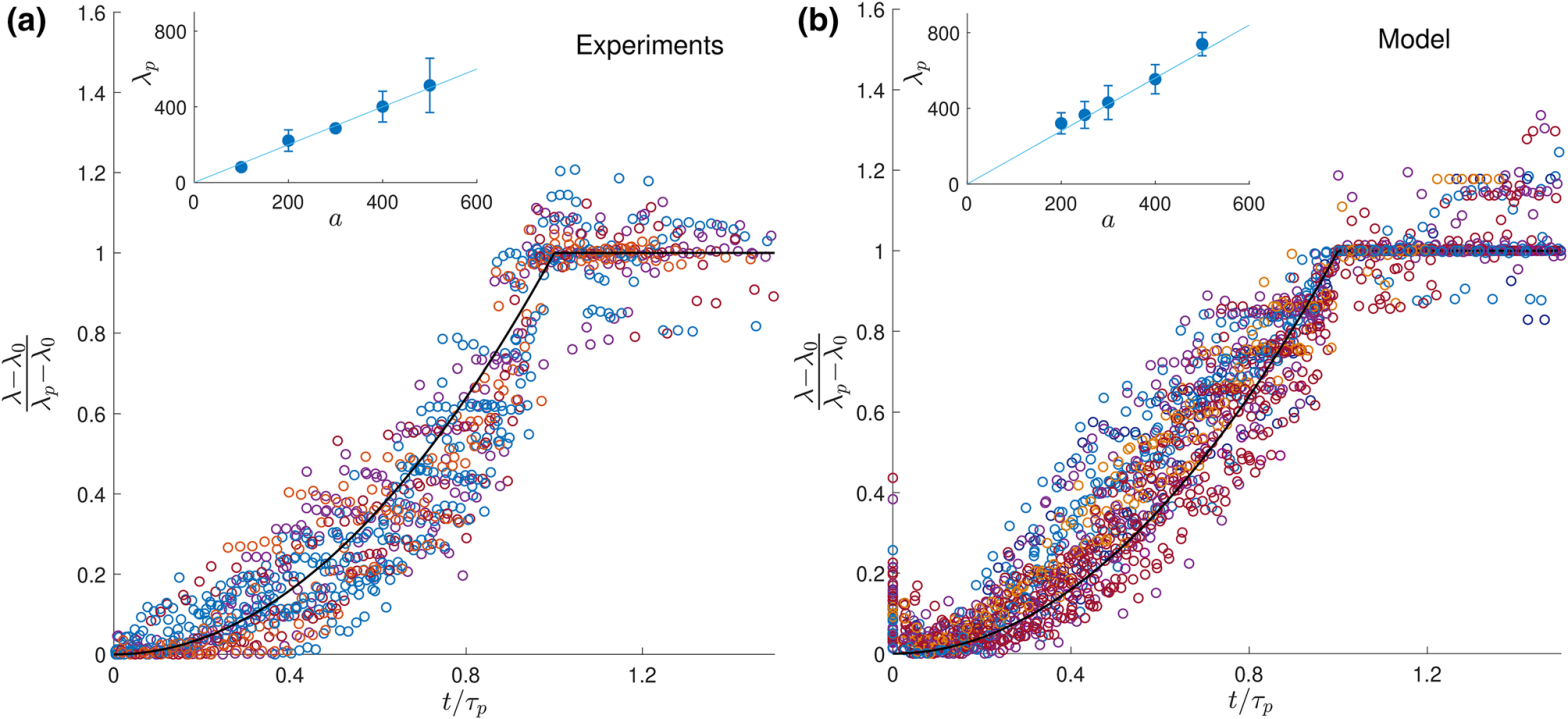
Time evolution of the wavelength in experiments (**a**) and model (**b**). In both cases, the wavelength $\lambda$ is rescaled using its initial ($\lambda _0$) and final values ($\lambda _p$) and is plotted as a function of the reduced time, $t/\tau _p$, where $\tau _p$ is the time at which $\lambda$ plateaus. Colours correspond to different confinement sizes. The black line is $y=x^2$ for $x<1$ and $y=1$ after. Insets show final wavelengths scaling linearly with channel size, with error bars showing standard deviation.

In order to quantify the dynamics of the emergent patterning, we measure the wavelength $\lambda$ of the plumes, defined as the average distance between neighbouring plumes, as a function of time. We locate the plumes in the experimental data by finding peaks in the intensity profile averaged along *z*. While we sometimes observe short-lived smaller plumes forming later in the experiments, we focus on the larger ones by defining an intensity threshold for the measured peaks (see details in Supplementary Material). Depending on the size of the channel, we observe between a few tens of plumes to two plumes at late times. As shown in Fig. 2a, the wavelength $\lambda (t)$ starts from an initial value $\lambda _0$, grows, and this growth accelerates until it reaches a plateau value at $\lambda _p$, after a time denoted by $\tau _p$. Remarkably, this final wavelength is found to scale linearly with the size of the capillary (Fig. 2a, inset). We note that in traditional convection, it is common for external length scales, including those due to confinement, to set the late-time convection wavelength^37^. Plotting the normalised wavelength $(\lambda -\lambda _0)/(\lambda _p-\lambda _0)$ as a function of the reduced time $t/\tau _p$ exemplifies the two regimes of the wavelength time evolution: monotonic growth followed by a plateau, as shown in Fig. 2a.

**Figure 3:**
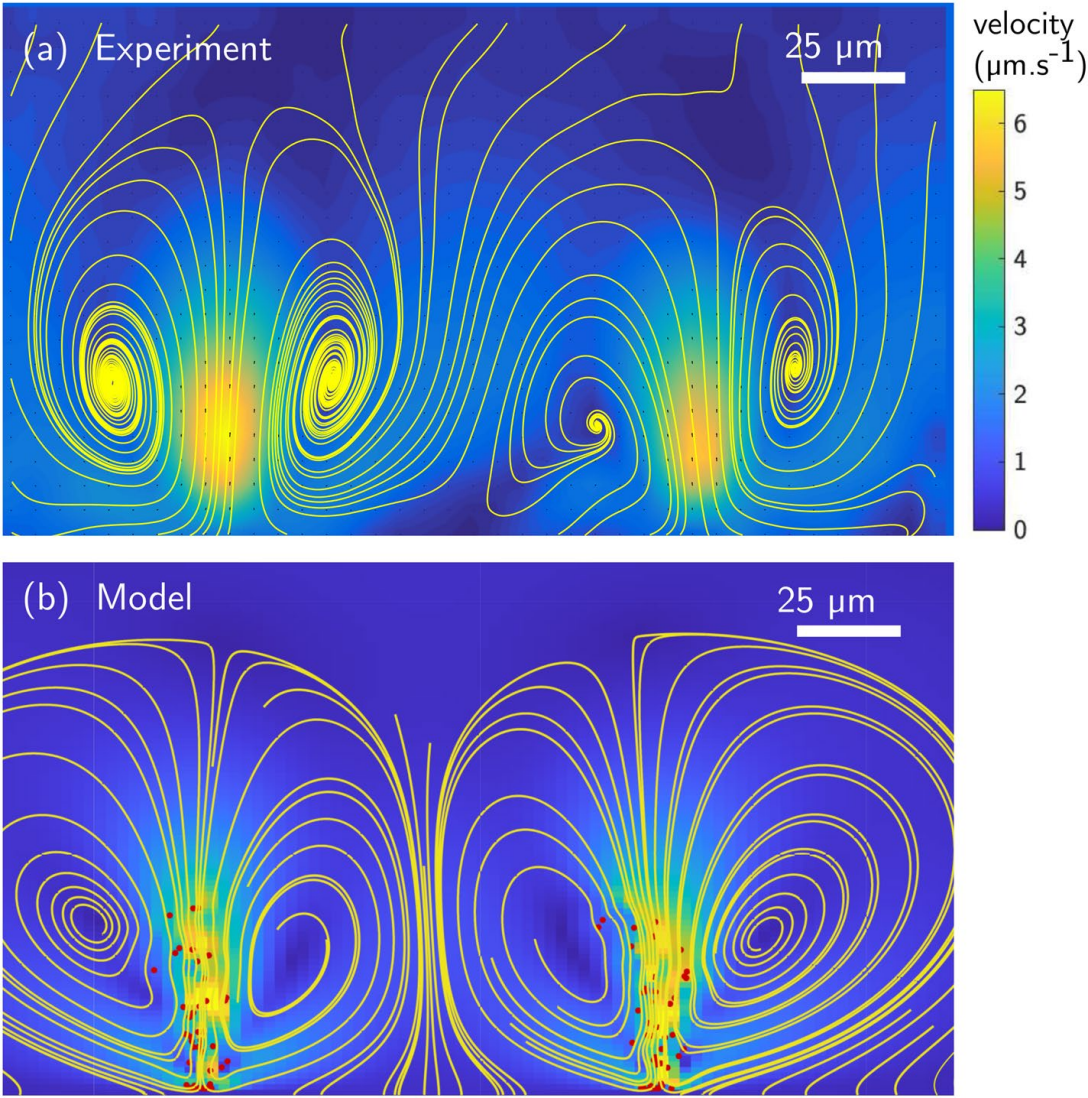
Convection cells created by two plumes in a $200~\upmu \mathrm{m}$ glass capillary. (**a**) Experimental measurements showing magnitude (colormap) and streamlines of the flow field averaged over one minute. (**b**) Convection cells and flow obtained with the theoretical model, with red dots representing the instantaneous location of the swimmers.

We next use PIV to measure the flow field and plot the streamlines and flow magnitude in Fig. 3a. Plumes are generated as soon as the characteristic flow velocities induced by the layer of accumulating bacteria are of the same order of magnitude as the downward velocity of the swimmers. When the wavelength becomes on the order of the channel width, hydrodynamic interactions with the walls screen the cellular flows and plumes stop growing, resulting in stable convection cells of size comparable to the channel.

### Hydrodynamic singularities model

To further understand the mechanism for the instability and its time evolution, we develop a theoretical model which successfully describes the emergence of collective behaviour and the formation of plumes in the suspension. We model AMB-1 as pusher swimmers^31, 40^. Each free-swimming bacterium is modelled as two opposite regularized point forces (known as stokeslets^41^) of strength $s_0$ separated by a distance $l_0$, taken as the characteristic size of the swimmer^42^ (see Supplementary Material and movies SM4 and SM5). The force-free swimmers are confined in a channel with square section *a* and aspect ratio 10 with periodic boundary conditions along the *x* direction (see Fig. 1a), and are subject to both steric and hydrodynamic interactions^20^. Magnetic interactions between swimmers are negligible compared to those with the external field, and are therefore neglected. We assume that the swimmers remain aligned with the external magnetic field and always tend to swim preferentially in the negative *z* direction at a constant speed $-{u} _0\hat{z}$. However, their speeds are modified by the dipolar flow fields induced by the other swimmers and by hydrodynamic interactions with walls. Our model investigates the system for values of the magnetic field above the critical field for bacterial alignment, consistent with what was done in the experiments. The model is non-dimensionalised using characteristic length scale $l_0$, velocity $u_0$ and dynamic viscosity $\mu$. For comparison with experiments, we use a characteristic bacterium size $l_0 = 5\, {\upmu \mathrm{m}}$^32^. Steric interactions are included using soft pair-repulsive forces^43^ between the centres of each bacteria. These interactions decay as *A*/*r* and, to speed up computation times, are only taken into account for bacteria closer than $2l_0$ and generate a speed with upper bound $5 u_0$. In addition to bacterium-bacterium interactions, hydrodynamic interactions also occur between bacteria and channel walls. We include the two walls normal to the magnetic field, $z_-$ and $z_+$, and use the method of images for regularized stokeslets (algebraic regularization function with a core of size $\delta = 0.3 l_0$) in order to enforce the no-slip boundary condition on the walls exactly (see Ref.^44^ and Supplementary Material). The image system for a stokeslet consists of a stokeslet, an irrotational dipole and a stokeslet dipole^45^ with adequate regularization. As a result, the position ${\varvec{x}}^{(j)}$ of the $j^{th}$ bacterium evolves with its velocity, ${\varvec{u}}^{(j)}$, sum of its intrinsic swimming speed $- u_0 {\varvec{e}}_z$, the hydrodynamic and steric interactions with other bacteria, denoted ${\varvec{u}}_{\mathrm{hydro}}$ and ${\varvec{u}}_{\mathrm{steric}}$ respectively, and the flow created by interactions of all swimmers with the walls, ${\varvec{u}}_{\mathrm{wall}}$, leading to

1 $$\begin{aligned} \frac{ {\mathrm {d}} {\varvec{x}}^{(j)}}{{\mathrm {d}} t} = {\varvec{u}}^{(j)} = - u_0 {\varvec{e}}_z + \sum _{i=1}^N {\varvec{u}}_{\mathrm{wall}}^{(j)}({\varvec{x}}^{(i)};{\varvec{x}}^{(j)}) +\sum _{i \ne j} \left[ {\varvec{u}}_{{\mathrm{steric}}}^{(j)}({\varvec{x}}^{(i)}-{\varvec{x}}^{(j)}) +{\varvec{u}}_{\mathrm{hydro}}^{(j)}({\varvec{x}}^{(i)}-{\varvec{x}}^{(j)})\right] . \end{aligned}$$

Our simulations have *N* swimmers, with *N* ranging from 500 to 8000, with different values of *N* used for each channel size *a*. Bacteria are initially distributed uniformly in the channel and their position is then integrated explicitly from Eq. (1) using the dimensionless time step $${\mathrm {d}}t = 0.5$$. Swimmers are confined laterally in the channel by ensuring that, after each time step, they remain inside the walls along $$y_+$$ and $$y_-$$, i.e. $$ -a/2< y^{(i)} < a/2$$. Note that in practice this constraints is rarely invoked in our simulations because hydrodynamic interactions between swimmers in the (*x*-*z*) plane are attractive and thus quickly lead to clustering within the channel. In the simulations, we take the bacteria to have identical swimming speed and therefore identical effect on the surrounding fluid. To account for the heterogeneity of the population, simulations were also carried out with bacteria velocities normally distributed around $$u_0$$ with standard deviation $$u_0/3$$ and we obtained the same plume patterns and dynamics.

**Figure 4:**
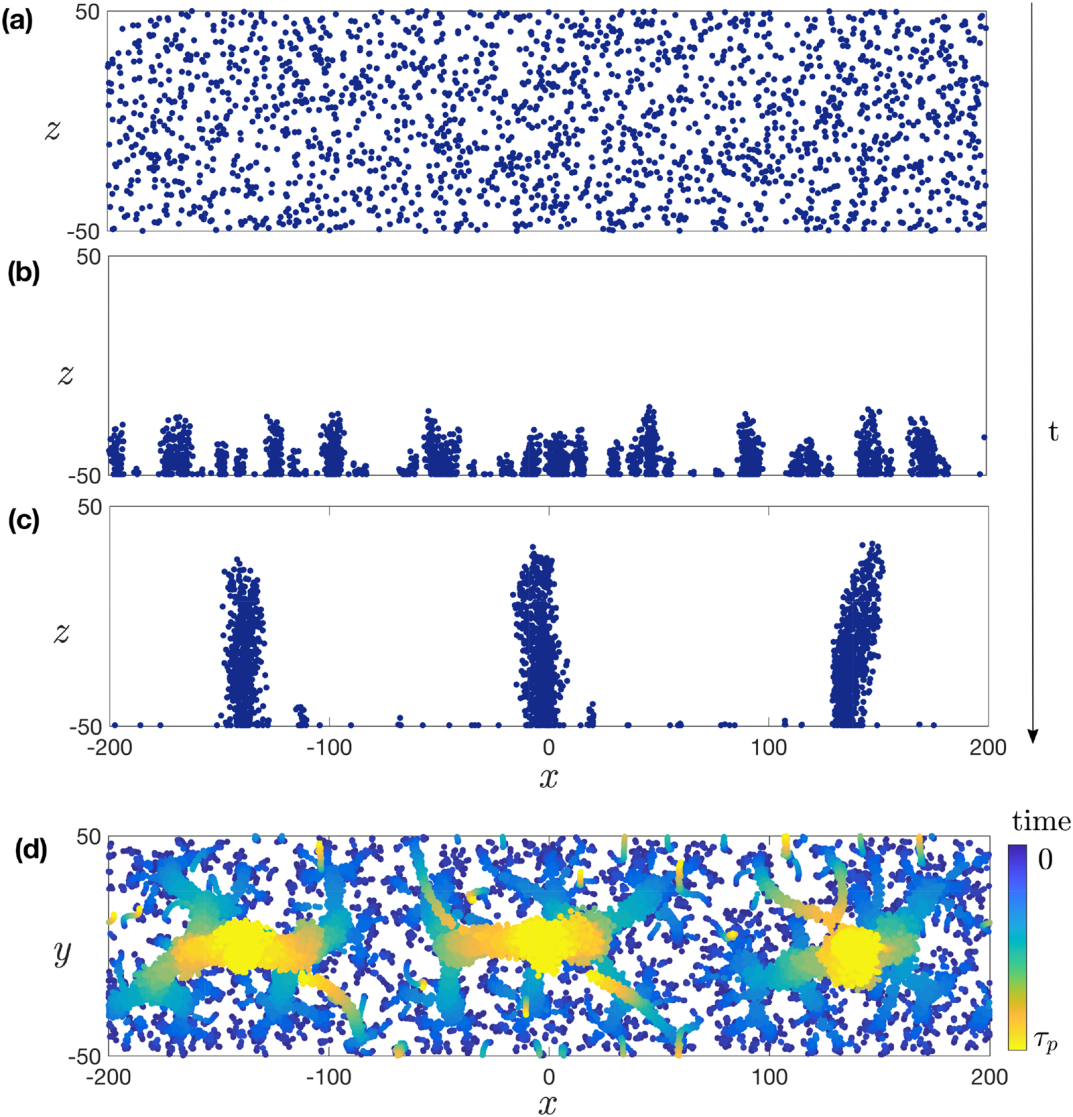
(**a**) Growth of plumes in the model starting from randomly distributed bacteria. (**b**) Swimmers accumulate at the wall and cluster due to hydrodynamic interactions. (**c**) The flows induced by the swimmers are sufficiently strong to detach some bacteria from the wall, leading to elongated plumes. (**d**) $x-y$ plane view showing the time-lapse evolution of the swimmers positions, from initially random (dark blue) to plumes in a steady flow state (light yellow) (dimensionless units).

The dynamics of the convective cells in the numerical model is illustrated in Fig. 4. Similar to the experimental observations, the randomly distributed bacteria (Fig. 4a) first swim in the $-z$ direction and accumulate at the wall (Fig. 4b). Neighbouring wall-bound bacteria experience attractive interactions and start clustering. The origin of this attraction lies in the hydrodynamic interactions between the bacteria and the wall. When a swimmer is located at the no-slip wall, the point force closer to the surface nearly cancels with its image, leaving only a stokeslet pushing the surrounding fluid away from the wall^22^. In other words, a bacterium stuck near a surface imposes a net force away from the wall on the surrounding fluid. Due to mass conservation, the flow created by this force induces a secondary attractive flow along the wall and directed toward the swimmer. Attractive interactions disturb the uniform distribution of wall-bound swimmers and lead to clustering. Attractive interactions between point forces located near a surface have been described in the past in both physics^46^ and biology^22^, including for MTB^31^. Similar hydrodynamic clustering has also been observed for *Thiovulum majus* (*T. majus*), a larger almost-spherical bacterium, swimming near walls^23^. While the mechanism for the initial attraction to the surface is the same, the evolution of the two systems differ significantly. The back-flow generated by the MTB in our experiments is strong enough to detach some of the swimmers and advect them away from the surface. Additionally, the *T. majus* bacteria in Ref.^23^ remain bound to the surfaces, possibly due to stronger interaction with the surfaces or because of their spherical shape, and form two-dimensional rotating crystals. In contrast, for both our experiments and model, detached MTB cells form plumes (Fig. 4c) which in turn generate stronger attractive flows, resulting in plume-plume attraction and merging. Finally, the increasing number of swimmers within each plume leads to the formation of elongated vertical plumes. This instability is predicted to occur at any bacterium concentration, as any two swimmers at the wall will experience attraction. Besides, cell detachment from the wall can occur for clusters of as little as three swimmers, which means that in our model, we predict no critical concentration for the instability. However, for very dilute systems, the timescale for plume formation becomes very long, and exceeds the time of our observations. The time-sequence from initial swimmer distribution to final plumes is illustrated in Fig. 4d with time-lapse representation of the swimmers position within the $x-y$ plane perpendicular to the plumes. Ultimately, a steady flow state is reached when plumes are separated by a length scale similar to the channel height, creating flows with convection cells similar to experimental observations (Fig. 3b). Note that, beyond the final state, we find a good qualitative agreement between the flow structures created by the plumes in our experiments and in the numerical model, as further detailed in the Supplementary Material.

To further compare the experimental data with the model, we study the dynamics of the plume wavelength, $\lambda$. In order to compute the wavelength, a histogram of the bacteria positions is obtained for each time point in the simulation. For consistency with experimental data analysis, plumes are identified where a peak is greater than 1/5 of the highest peak. Similarly to experiments, simulations reveal a sharp change in wavelength evolution, corresponding to a transition from accelerating growth to a plateau in the wavelength. Rescaling the wavelength leads to collapse of the numerical results for various concentrations and system sizes in excellent agreement with the experimental data (Fig. 2). In particular, the late-time wavelength scales with the size of the channels in both cases (we obtain $\lambda _p \sim a$ in the experiments and $\lambda _p\sim 1.4a$ for the simulations). Our simulations support an intuitive physical explanation for the existence of a plateau value wherein the transition occurs when plumes interact with their hydrodynamic images on the opposing wall, which limits the range of the flow created by all swimmers in a plume and thereby plume growth. The confinement thus sets the limit for the accelerating merging of the plumes. To validate this hypothesis, we carried out simulations without an upper wall (i.e. unconfined) and obtain, for sufficiently large concentrations, uninterrupted growth until only a single plume emerges (see Supplementary Material). We note that the presence of walls parallel to the magnetic field, $y_-$ and $y_+$, is neglected in simulations and would further slow the growth of the biggest plumes due to additional flow confinement.

**Figure 5:**
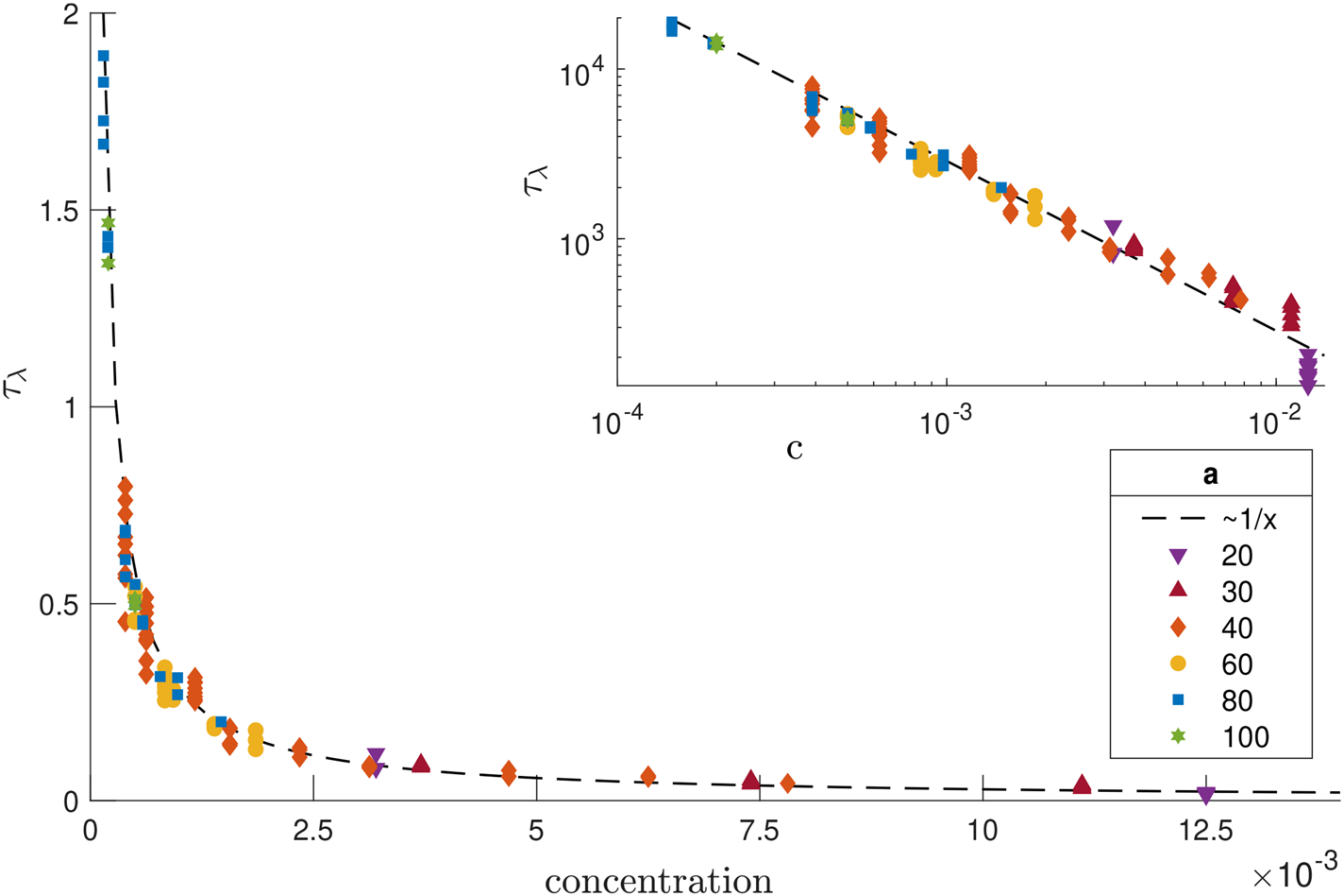
Characteristic time for the growth of the wavelength, $\tau _{\lambda }$, as a function of the concentration, *c*, in our simulations (both quantities are plotted in dimensionless units). The time scale $\tau _{\lambda }$ is obtained using a least-square quadratic fit of the wavelength of the form $\lambda (t) /a = \lambda _0 / a + (t/\tau _\lambda )^2$. The dotted line is a fit to an inverse scaling as $\tau _\lambda \propto 1/c$. Inset: same plot in log-log scale.

Focusing on wavelength dynamics before reaching the plateau regime, $\lambda (t)$, we observe some scatter in the curves of Fig. 2 for both experiments and in our model, showing that the precise wavelength growth dynamics vary with the channel size *a* and the cell concentration (see Supplementary Material). This shows that the time to reach the plateau $\tau _p$ does not fully govern the early dynamics of the system. Indeed, we identify two regimes in the simulations, one of accelerating growth and one of approximately constant growth. Their relative duration sets the shape of the $\lambda (t)$ curves in Fig. 2. In the first one, some bacteria have not yet reached the $z_-$ region of the channel as the first plumes start forming. The duration of this first regime can be estimated by computing the average of the lowest *z* value previously reached by each bacterium, and we see that it is higher than for free swimming bacteria due to repulsive hydrodynamic interactions in the *z* direction. Increasing swimmers concentration in the $z_-$ region accelerates plume growth (see Fig. 5), and this first regime coincides with an increasing rate of growth of the wavelength, as further detailed in the Supplementary Material.

At later times, all bacteria are in the $z_-$ region of the channel. If the flow created by a plume was the sum of all flows generated by individual bacteria at the wall, then $\lambda \propto t^{1/2}$, due to localised hydrodynamic forcing (Stokeslets) near surfaces interacting as $r^{-2}$, which matches neither the experiments nor the simulations. However, when in a plume, swimmers are advected away from the wall and their contribution to the flow is modified and decays more slowly. Because of this, the flow created by a plume does not scale linearly with the number of swimmers in the plume, which leads to more complex dynamics without a single scaling law. Plume-plume interactions depend on the position of individual swimmers relative to the wall and hence on the density profile of plumes. We find that plumes are systematically denser near the wall, with some bacteria sticking on it (see Supplementary Material). Integrating the inverse of the flow velocity in the *x* direction from simulations of a single stratified plume yields an approximation for the merging time of two plumes, given their initial separation $x_0$. Due to mass conservation, for a given system where plumes gradually merge, $x_0$ scales with the number of swimmers in a plume, $N_p$. We intuitively expect the merging time for two plumes to be linear with $N_p$, and this is observed for sufficiently large swimmer concentrations, corresponding to the concentrations used in our simulations to match experimental observations. In contrast, lower concentrations lead to longer merging times for the plumes and therefore smaller plumes, as observed both in experiments and simulations. In the regime where the bacteria have reached the $z_-$ part of the channel, the wavelength varies linearly with time. This linear scaling is confirmed by simulations where all bacteria were initially positioned on the $z_-$ surface. In that case, the wavelength depends linearly on time from the beginning of the simulation (see Supplementary Material). The time scale of the dynamics $\tau _p$ is therefore larger than the time for a single bacterium to swim to the wall but smaller than the time for bacteria at the wall to attract each other on distance comparable to the cross section *a*. To quantify the influence of the concentration on plume growth in our model, we estimate a characteristic time scale, $\tau _\lambda$, for wavelength growth obtained from a quadratic fit of the wavelength $\lambda (t)$, with results shown in Fig. 5. We see that $\tau _\lambda$ scales as the inverse of the concentration, which shows that the dynamics of the system are governed by the plume-plume merging time scaling as $N_p$, as described above. Overall, the nonlinear shape of the wavelength dependence on time can thus be explained by the time it takes for swimmers to reach the plumes region and the advection of bacteria away from their hydrodynamic images, which tends to increase their contribution to the flow and thus the attractive interactions between plumes.

In summary, we have identified experimentally a new type of collective motion, bacterial magneto-convection. MTB driven by an external magnetic field start by accumulating at the surface of a microfluidic channel. The initial random distribution of bacteria is unstable due to hydrodynamic attraction between swimmers, which cluster and form dynamic plumes. The resulting self-sustained flow resembles convection cells, with a late-time characteristic size linearly dependent on the confinement length scale, with the crucial difference that here no external forces are applied to the system, only external torques aligning the cells with the external field. A model based on hydrodynamic singularities and their images describes interactions between bacteria and with channel walls and successfully reproduces the growth and long-time dynamics of the interacting plumes. We expect this model to be useful in understanding other observed collective motions of magnetotactic bacteria as well as any confined swimmers oriented by external fields, leading to a better understanding of the respective role of swimmer-swimmer interaction and external cues. Since bacterial magneto-convection represents a new class of collective behaviour resulting only from the balance between hydrodynamic interactions and external alignment, it helps elucidate the synergy between particle-particle interactions and external cues and can be compared to other systems of biased biological or artificial swimmers to shed a light on their own collective dynamics.

## Methods

Bacteria are grown in a liquid medium with controlled nutrients and O$_2$ conditions, following Ref.^47^. To ensure that most of the bacterium population remains magnetotactic and motile, we use a racetrack selection method^39^, keeping only bacteria that swim through a filter while guided by an external magnetic field. This also selects bacteria with a preferred polarity. Before the experiments reported in the present study, we measured the magnetic moment of bacteria grown in the same conditions and obtained $\mu =10^{-15}\pm 4\times 10^{-16} {\mathrm{A\,m}^2}$ (mean ± standard deviation)^48^. High concentrations of motile bacteria are then obtained by centrifugation ($1.5 {\mathrm{ml}}$, $2000\, {\mathrm{rcf}}$, $2\,\mathrm{min}$) and subsequent re-dispersion in approximately 10% of the supernatant. After this procedure, a majority of the obtained bacteria are motile and magnetic, however the solution also contains non motile and dead bacteria. We fill the glass capillaries (Vitrotubes, VitroCom, New Jersey, USA) by capillarity with a drop of the concentrated suspension. Both sides of the capillary are then sealed by placing them on a support with two cells filled with mineral oil, to avoid evaporation.

Two Helmholtz coils are mounted on the platform of a microscope (Nikon eclipse E200) to generate a uniform magnetic field normal to the capillary tube. We verify that the magnetic field was indeed uniform on the whole length of the capillary and calibrated the set up to obtain fields ranging from $1 \times 10^{-4} \,{\mathrm{T}}$ to $20 \times 10^{-4}\, {\mathrm{T}}$. Images are then acquired with a CCD camera (Allied Vision Technology GE680) at $10\,{\mathrm{fps}}$. The images are then analysed through PIV^49^, using bacteria as tracers. While motile magnetic bacteria accumulate at the surface of the capillary and in the plumes, the presence of non-motile bacteria enables us to observe the flow field in the capillary throughout the experiments, and obtain streamlines in the channel.

## Supplementary information

Below is the link to the electronic supplementary material.

Supplementary Information 1.

Supplementary Information 2.

Supplementary Information 3.

Supplementary Information 4.

Supplementary Information 5.

Supplementary Information 6.

## Data Availability

The data that support the findings of this study as well as the Matlab code (for image analysis and simulations) are available from the corresponding author upon reasonable request.
