# Supplementary material for: Self-organisation and convection of confined magnetotactic bacteria
Source: Sci Rep. 2020 Aug 11;10:13578. doi: 10.1038/s41598-020-70270-0 (PMC7419309; doi:10.1038/s41598-020-70270-0)
Supplement: Supplementary file 6 — Supplementary Information 6. [file 41598_2020_70270_MOESM6_ESM.pdf]

Self-organisation and convection of confined magnetotactic bacteria Supplementary Material

Albane Théry,[1,2] Lucas Le Nagard,[2,*] Jean-Christophe Ono-dit-Biot,[2,*]
Cécile Fradin,[2] Kari Dalnoki-Veress,[2,3,†] and Eric Lauga[1,‡]

[1]*Department of Applied Mathematics and Theoretical Physics, University of Cambridge, Cambridge CB3 0WA, United Kingdom.*

[2]*Department of Physics and Astronomy, McMaster University, 1280 Main St. W, Hamilton, ON, L8S 4M1, Canada.*

[3]*UMR CNRS Gulliver 7083, ESPCI Paris, PSL Research University, 75005 Paris, France.*

(Dated: July 16, 2020)

DATA ANALYSIS

In order to quantify the dynamics of the merging plumes from the sequence of experimental images, we average in the z direction the intensity of the z_- part of the image where the plumes originate. This average is taken over a region of size comparable to the growing plume height, approximately $z = 50$ μm. This allows us to detect peaks in the intensity profile corresponding to plumes. In order to adjust for each experiment the parameters for peak finding (namely the minimal height and width of a peak as well as the minimal distance between neighbouring peaks) we check manually that the obtained peaks match the position of the plumes in the data (Fig. S 1a). The wavelength at a given time is then defined as the mean distance between neighbouring intensity peaks. To avoid fluctuations in the wavelength due to peak detection, we use the moving median of the computed wavelength over 20 images (2 s) (Fig. S 1b).

In Fig. S 2 we present the raw data for the wavelength as a function of time for different channel sizes. This data is then rescaled in Fig. 2 of the main article to show the two distinct regimes of plumes time-evolution, growth and plateau.

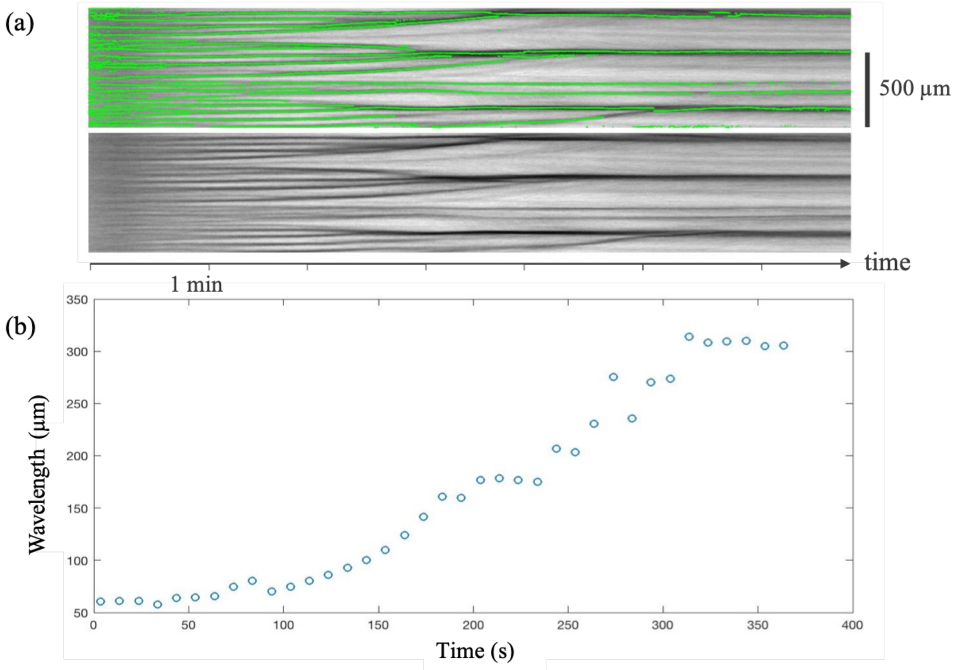

FIG. S 1: (a): Space-time plots showing the time evolution of intensity in the z_- region of a 500 μm capillary. In the top plot, green points depict peaks in intensity which correspond to plumes as detected numerically (b) Time-evolution of the wavelength as obtained from the above data.

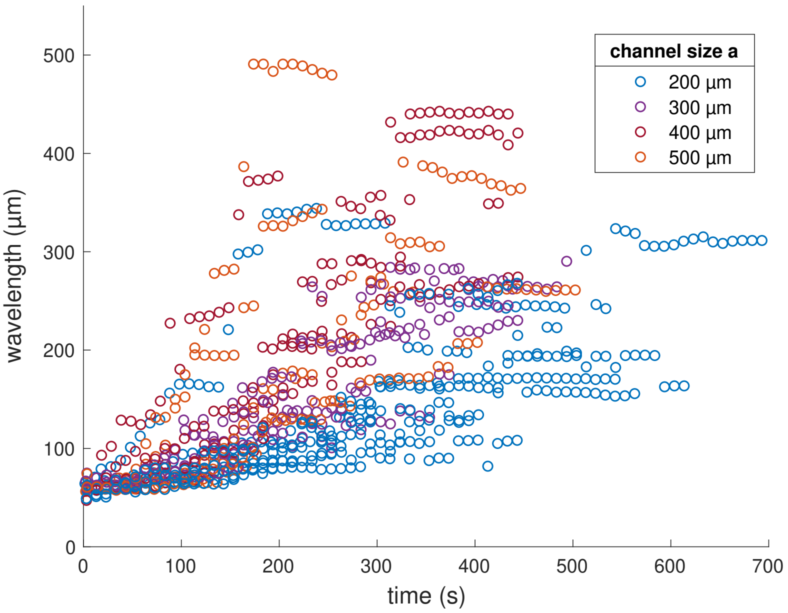

FIG. S 2: Plume wavelength (μm) as a function of time (s) for different experiments with capillary channel sizes varying from 200 μm to 500 μm

ADDITIONAL EXPERIMENTAL PARAMETERS

We identified several key parameters that govern the dynamics of bacterial magneto-convection. While elucidating quantitatively their role would require a different experimental set-up, we present here some experimental findings that shed some light on their influence over the system dynamics.

Magnetic field

To understand the influence of the magnetic field strength in the plume formation and growth, we experimentally vary the field strength in a single capillary tube sample. We switch on the field, let the plume evolve for 200 s, then switch the field off. Once the plumes disappear, the experiment is re-started using a different field strength with the same population. We check the validity of this procedure by re-using the same field after a few such cycles and ensuring that wavelength time-evolution is unchanged (Fig. S 3a).

We find that there exist a critical field below which magneto-convection does not occur. This critical field is strongly dependent on the bacterial population and concentration, and we measured values ranging from 0.25 to 1 mT.

While we were not able to investigate fully the critical behaviour, we observe that below this threshold value, no plumes are formed. Slightly above this value, we observe smaller

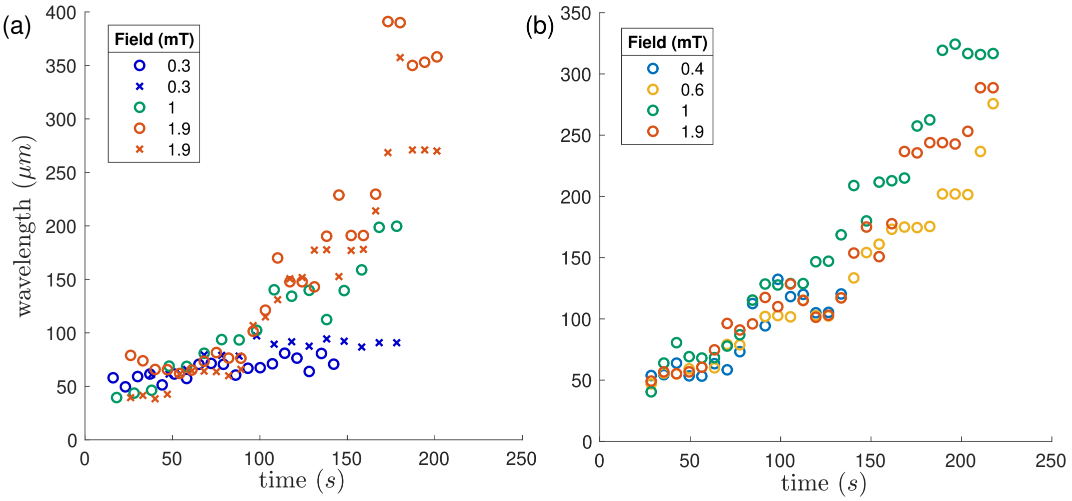

FIG. S 3: Time evolution of the wavelength for two different 400 μm capillaries, changing the strength of magnetic field in a single population (a) Below the critical field (at 0.2 mT), no plumes appear (no wavelength is measured, not shown), close to the critical field (0.3 mT), small plumes appear but do not grow, and above the critical field, plume time-evolution becomes independent of the magnetic field (b) Above the critical magnetic field, the wavelength is independent of the field strength

plumes that do not grow, as presented in Fig. S 3a. Above this value, the coarsening is independent of the magnetic field. Thus, beyond some critical magnetic field strength the speed of plume growth is independent of the field (Fig. S 3b). We attribute this to a field that is strong enough to align the bacteria.

Except for the data presented in Fig. S 3, all the experiments were performed above the critical field (2 mT) to rule out any influence of the field strength on the plume growth.

Orientation of the bacteria

To further validate the assumption that the bacteria remain aligned with the magnetic field in the high field regime, we derive here the theoretical orientation distributions of individual bacteria characterized by a magnetic moment $\vec{\mu}$ placed in a magnetic field $\vec{B}$ at room temperature ($T = 293\,\mathrm{K}$). Magnetotactic bacteria have orientation statistics that can be described by a paramagnetic model, and their orientation distributions therefore follow Boltzmann statistics [1]. Considering a magnetic field $\vec{B}$ along the z axis, α the angle between $\vec{B}$ and $\vec{\mu}$, and ϕ the azimuthal angle, the probability of a particular orientation is given by

$$p(\alpha, \phi) = \frac{e^{-E(\alpha,\phi)/k_B T}}{Z}, \tag{1}$$

where $E(\alpha, \phi) = E(\alpha) = -\mu B \cos\alpha$ is the magnetic interaction energy, k_B is the Boltzmann constant and $Z = \int_0^{\pi}\int_0^{2\pi} e^{\mu B \cos\alpha / k_B T} \sin\alpha \; d\phi d\alpha = \frac{4\pi k_B T}{\mu B} \sinh\left(\frac{\mu B}{k_B T}\right)$ is the partition function. In the following, we write $A = \frac{\mu B}{k_B T}$. This model allows us to calculate the probablility for the cells to be at an angle $a \leq \alpha \leq b$ from the field $\vec{B}$

$$p(\alpha \in [a, b]) = \frac{e^{A\cos a} - e^{A \cos b}}{2\sinh(A)}. \tag{2}$$

We independently measured the value of μ on bacteria grown in the exact same conditions. We obtained $\mu = 10^{-15} \pm 4 \times 10^{-16}\,\mathrm{A\,m^2}$ (mean $\pm$ standard deviation) and reported the full results elsewhere [2]. From this, we compute and report in Fig. S 4 the theoretical discrete orientation distributions obtained in four limiting cases ($B = 0.2\,\mathrm{mT}$ or $B = 2\,\mathrm{mT}$ and $\mu = 10^{-15}\,\mathrm{A\,m^2}$ or $\mu = 5 \times 10^{-16}\,\mathrm{A\,m^2}$). There is no significant difference between the two distributions computed for $B = 2\,\mathrm{mT}$ (Fig. S 4a and d), indicating that all the bacteria in the population are likely to behave in a similar way in the high field regime. Both histograms are narrow and reach their maxima for $\alpha < 5°$. In striking contrast, distributions computed for $B = 0.2\,\mathrm{mT}$ (Fig. S 4b and e) are broad and extend to significantly larger values of α, illustrating the decay of the alignment at low enough fields.

Furthermore, the average value of $\cos\alpha$ can be derived from Eq. 1 and is given by the Langevin function, as expected in the paramagnetic model framework:

$$\langle \cos\alpha \rangle = \coth(A) - \frac{1}{A}. \tag{3}$$

The graph of $\langle \cos\alpha \rangle$ as a function of B is presented in Fig. S 4 for $\mu = 10^{-15}\mathrm{A\,m^2}$ and $\mu = 5 \times 10^{-16}\mathrm{A\,m^2}$, corresponding respectively to the mean and lower bound values of μ expected for our bacteria population. The value of $\langle \cos\alpha \rangle$ is a quantity of particular interest, as $u_0\langle \cos\alpha \rangle$ is in first approximation the average magnitude of the bacteria speed (magnitude u_0) along the magnetic field axis. In the high field regime used in our experiments ($B = 2\,\mathrm{mT}$), $\langle \cos\alpha \rangle \simeq 1$, which validates the assumption that the bacteria can only swim along the applied magnetic field. We also note that $\langle \cos\alpha \rangle$ starts to become visibly lower than 1 for fields lower than $0.4\,\mathrm{mT}$ and drops significantly for fields lower than $0.2\,\mathrm{mT}$, which is consistent with our experimental observation that there is a critical field around these values, below which no plumes can form and grow, due to an insufficient alignment with the field.

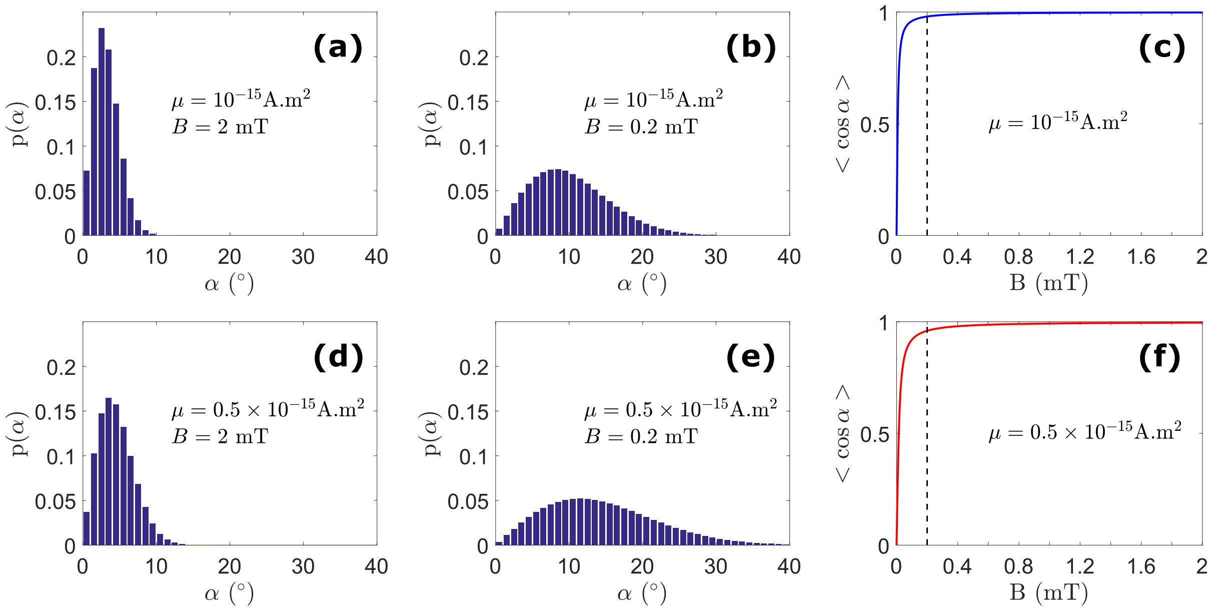

FIG. S 4: Theoretical orientation statistics obtained from the paramagnetic model.(a, b) Discrete orientation distributions for $\mu = 10^{-15}\,\mathrm{A\,m^2}$ and (a) $B = 2\,\mathrm{mT}$ and (b) $B = 0.2\,\mathrm{mT}$. (c) Relationship between $\langle \cos\alpha \rangle$ and B for $\mu = 10^{-15}\,\mathrm{A\,m^2}$. (d, e) Discrete orientation distributions for $\mu = 0.5 \times 10^{-15}\mathrm{A\,m^2}$ and (d) $B = 2\,\mathrm{mT}$ and (e) $B = 0.2\,\mathrm{mT}$. (f) Relationship between $\langle \cos\alpha \rangle$ and B for $\mu = 0.5 \times 10^{-15}\,\mathrm{A\,m^2}$. The vertical dash line plotted at $B = 0.2\,\mathrm{mT}$ in (c) and (f) is a guide to the eye illustrating the lower limit of the high field regime, in which the perfect alignment hypothesis holds true.

MODEL

Simulation details

In our simulations, we consider N swimmers each located at $\boldsymbol{x_k} = (x_k, y_k, z_k)$, $1 \leq k \leq N$. Each swimmer consists of two Stokeslets of strength $+s\boldsymbol{e}_z$ and $-s\boldsymbol{e}_z$ located respectively at positions $(x_k, y_k, z_k + l_0/2)$ and $(x_k, y_k, z_k - l_0/2)$. To implement the no-slip boundary conditions on both walls ($z_+ = a$ and $z_- = 0$), we use the image system of each Stokeslet, a set of singularities placed in a symmetric position with respect to the wall that cancel out the flow at the wall. For a singular Stokeslet of strength $\boldsymbol{s}$ orthogonal to a single wall, the image system as described in Ref. [3] is a Stokeslet of strength $-\boldsymbol{s}$, a Stokeslet dipole of strength $-2h\boldsymbol{s}$ and an irrotational source dipole of strength $2h^2\boldsymbol{s}$ where h is the distance between the bacterium and the wall, (Fig. S 5). We only include the first image of the swimmers on each wall, and ignore the additional reflections of the image systems.

To avoid singularities in the flow, we use regularised Stokeslets with a spatially distributed force rather than a Dirac distribution, as presented in Ref. [4]. We use a blob of size $\delta = 0.3l_0$ comparable to the size of a bacterium and the regularisation function of equation (18) in Ref. [4], namely

$$\psi_\delta(r) = \frac{15\delta^4}{8\pi(r^2 + \delta^2)^{7/2}}. \tag{4}$$

The regularised image system is composed of the same singularities but with different regularisation functions, and the corresponding flow is given in equation (21) of Ref. [4]. We consider the flow created at $\boldsymbol{x_e}$ by a Stokeslet at $s\boldsymbol{e_z}$ $\boldsymbol{x_s} = (x_k, y_k, z_k + l_0/2)$. The image

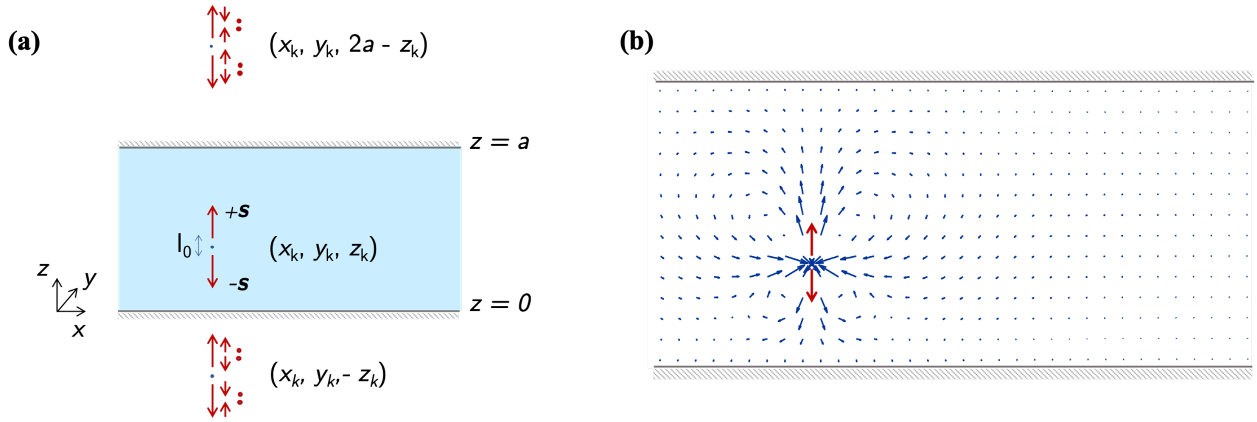

FIG. S 5: (a) Singularities for a swimmer (two Stokeslets) and its image systems (two Stokeslets, two Stokeslet dipoles and irrotational source dipoles) for one reflection on both walls. The red symbols represent the components of the singularities, arrows for point forces and dots for point sources. (b) Corresponding velocity vector field in a channel of size $a = 100$ with a swimmer at $z = 35$.

points of the Stokeslet on the lower and upper walls are $\boldsymbol{x_{im,L}} = (x_k, y_k, -z_k - l_0/2)$ and $\boldsymbol{x_{im,U}} = (x_k, y_k, 2a - z_k - l_0/2)$ respectively. We write $\boldsymbol{x} = \boldsymbol{x_e} - \boldsymbol{x_s}$, $\boldsymbol{x_L} = \boldsymbol{x_e} - \boldsymbol{x_{im,L}}$ and $\boldsymbol{x_U} = \boldsymbol{x_e} - \boldsymbol{x_{im,U}}$. The velocity is then given by

$$\begin{aligned}
\boldsymbol{u}(\boldsymbol{x_e}) = s\Big\{ & H_1(|\boldsymbol{x}|) + zH_2(|\boldsymbol{x}|)\boldsymbol{x} - H_1(|\boldsymbol{x_L}|) + z_L H_2(|\boldsymbol{x_L}|)\boldsymbol{x_L} \\
& - (z_k + l_0/2)^2 \left[D_1(|\boldsymbol{x_L}|) + D_2(|\boldsymbol{x_L}|)\boldsymbol{x_L}\right] \\
& + 2(z_k + l_0/2)\left[\left(H_2(|\boldsymbol{x_L}|) + z_L^2 \frac{H_2'(|\boldsymbol{x_L}|)}{|\boldsymbol{x_L}|}\right)\boldsymbol{x_L} + \left(z_L H_2(|\boldsymbol{x_L}|) + \frac{H_1'(|\boldsymbol{x_L}|)}{|\boldsymbol{x_L}|}\right)\boldsymbol{e_z}\right] \\
& - H_1(|\boldsymbol{x_U}|) + z_U H_2(|\boldsymbol{x_U}|)\boldsymbol{x_U} - (z_k + l_0/2)^2 \left[D_1(|\boldsymbol{x_U}|) + D_2(|\boldsymbol{x_U}|)\boldsymbol{x_U}\right] \\
& + 2(z_k + l_0/2)\left[\left(H_2(|\boldsymbol{x_U}|) + z_U^2 \frac{H_2'(|\boldsymbol{x_U}|)}{|\boldsymbol{x_U}|}\right)\boldsymbol{x_U} + \left(z_U H_2(|\boldsymbol{x_U}|) + \frac{H_1'(|\boldsymbol{x_U}|)}{|\boldsymbol{x_U}|}\right)\boldsymbol{e_z}\right]\Big\},
\end{aligned} \tag{5}$$

with H_1, H_2, D_1, D_2 regularisation functions given explicitly in Ref. [4]. The flow created by a swimmer is then obtained by adding the flow of the second Stokeslet of strength $-s\boldsymbol{e_z}$ at $\boldsymbol{x_s} = (x_k, y_k, z_k - l_0/2)$, which has the same expression as above but with $s \to -s$ and $z_k + l_0/2 \to z_k - l_0/2$.

The value of s is $6\pi\mu l_0 u_0$, with non dimensional value $s = 6\pi$. Simulations with heterogeneous bacteria population are made using a normal distribution for the downwards velocities, with mean u_0, standard deviation $u_0/3$. We cut negative values so that bacteria all swim in the $-z$ direction. Corresponding strength of hydrodynamic singularities $s = 6\pi\mu l_0 u_{0i}$ is then used to compute bacteria-bacteria interactions.

The time evolution of the system is obtained by computing the flow created on each swimmer by both its image and the other swimmers and their images. We then update the position of each swimmer using a forward Euler method with time step $\mathrm{d}t = 0.5$. The coordinates of each swimmers are stored in a $3 \times N$ matrix updated at each iteration. To obtain the flow and streamlines generated by the swimmers (see Fig. 3 of the main article), we use a 100×1000 grid in the channel and compute the velocity vector field.

Characterisation of plume structure

Plume-plume hydrodynamic interactions, and therefore the dynamics of the whole system, depend on the position of individual swimmers relative to the wall and hence on the density profile within each plume. Although we are not able to analyse the experimental structure of the plume, we can investigate in the model the density profile and local flow around plumes with different numbers of swimmers, as shown in Fig S. 6. We find that plumes of all sizes are denser near the wall, with some bacteria sticking to it. For the bacteria detached from the wall, the density decreases linearly from the bottom to the tip of the plume. This stratified structure corresponds to a balance between downward swimming and repulsive interactions between bacteria in the vertical z-direction.

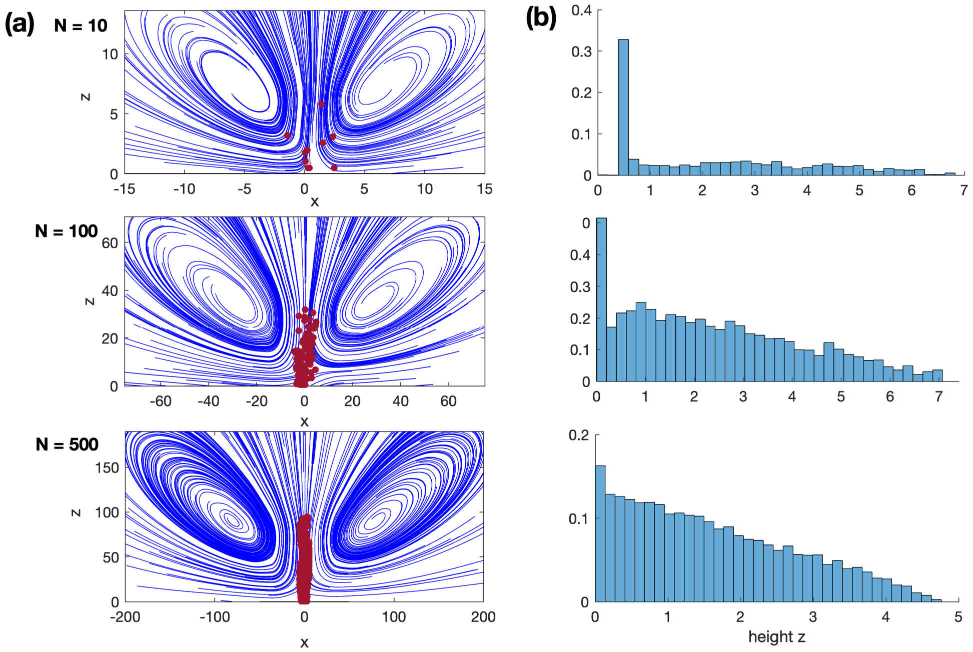

FIG. S 6: (a) Streamlines of the generated flow and (b) probability of the density for plumes with a varying number of swimmers $N = 10, 100, 500$

Short time dynamics

We investigate the short time dynamics of the system and we show that the convexity of the wavelength vs. time relationship observed in experiments and simulations, especially at the beginning of plume formation, originates from the time that it takes for bacteria to accumulate at the bottom of the channel.

At short times, the bacteria start at random in the channel. They then swim downwards and accumulate at the lower z_{-} wall. In this first regime, the local concentration in the plume region (close to the z_{-} wall) is increasing with time. As shown in the main text, higher concentrations lead to faster plume dynamics. We therefore expect wavelength growth to accelerate while the bacteria accumulate at the wall. This explanation is borne out by the observation that the wavelength is linear if all the bacteria start instead at the bottom (Fig. S 7). We also evaluate the time for all the bacteria to reach the lower region of the channel and show that it is the same as the characteristic time in which the wavelength growth is accelerating. This time for bacteria to accumulate is made longer by repulsive hydrodynamic interactions between swimmers along the z direction, as shown in Fig. S 7.

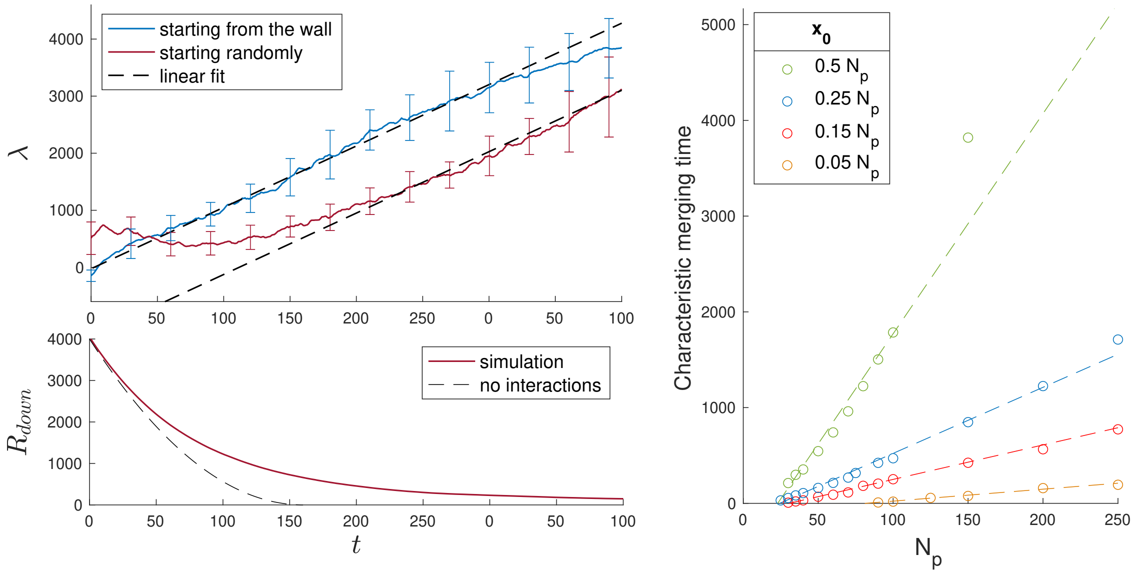

FIG. S 7: (a) Wavelength at the beginning of the simulations for bacteria starting at the wall (blue) and at random height (red) (we plot the mean of 20 simulations ± one standard deviation). The dashed line is a linear fit for the wavelength of the bacteria at the wall (top dashed line) and its translation used to compare to the random starting point case (bottom dashed line). (b) Time-evolution of the average of the lowest point reached by each swimmer in full simulations (red) and in a case with no hydrodynamic interactions (black dotted line) (c) Characteristic time for two plumes to merge depending on their size N_p and their starting distance x_0 (lines show a linear fit). Lower merging times correspond to higher concentrations. For lower concentrations, plume merging slows down and the wavelength is no longer convex, which is coherent with simulations

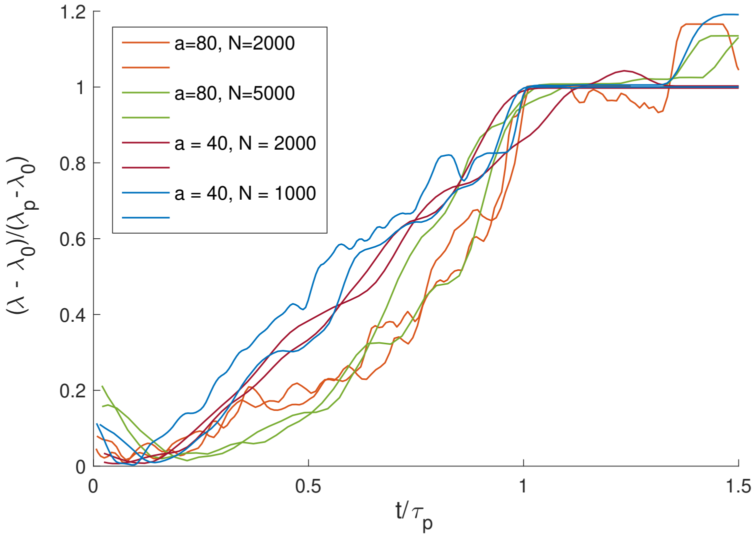

FIG. S 8: Rescaled wavelengths λ for different sets of channel sizes a and numbers of bacteria N (as in Fig. 2 of the main article). Scatter is caused in particular by the variation in the duration of the early-time regime relative to the time τ_p to reach plateau.

Due to the existence of a short-time regime, we expect the timescale of the early accelerating growth of the wavelength to have a different dependence on the system parameters (channel size a and number of swimmers N) than the time to reach the wavelength plateau, τ_p. For an identical number of swimmers, the bacteria will take longer to swim down for larger channels, and the first regime of accelerated growth will be longer relatively to τ_p. For identical channel sizes and a larger number of swimmers, repulsive hydrodynamic interactions in the z direction will make the absolute time for all bacteria to reach the z_- wall longer. Because of this variation of the early dynamics, there is some scatter when rescaling the wavelength as a function of time as in Fig. 2 of the main article: Fig. S 8 indeed shows that while identical parameters lead to similar wavelength dynamics, varying a and N modifies the variation for $\lambda(t)$ and leads to scatter.

The role of hydrodynamic images

To gain insight into the role of hydrodynamic image singularities in the plume dynamics, we also performed simulations ignoring the image system on the upper z_+ wall (Fig. S 9). We observe that, without the hydrodynamic images, the dynamics is faster. At high concentration, we find that at long-time the final state is a single plume, taller than the channel size. On the contrary, when the plumes interact with their images on the z_+ wall, the dynamics are slowed down. We see then in both simulations and experiments, that the channel width a sets the long term wavelength.

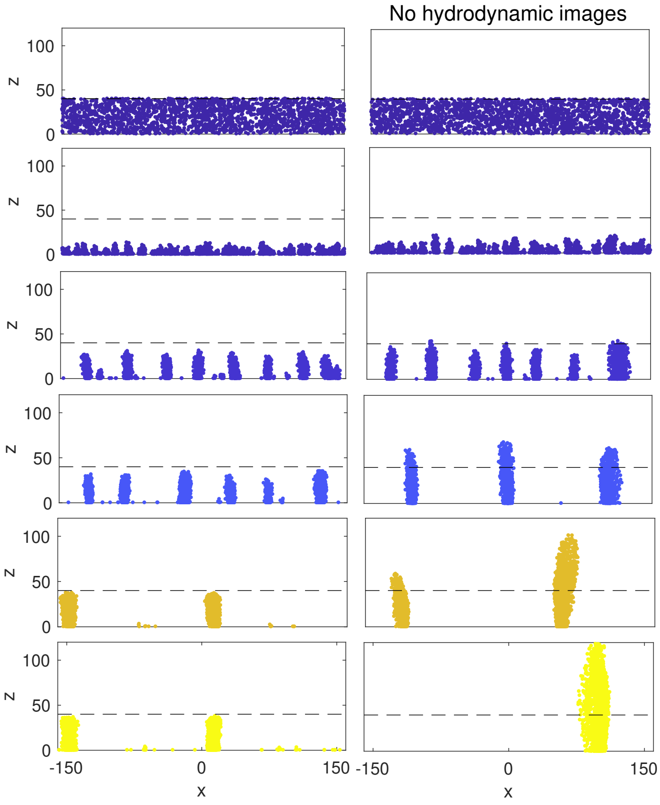

FIG. S 9: Dynamics of the plumes with (left) and without (right) their hydrodynamic images on the top z_+ wall. The channel size a is 40 with an aspect ratio 8. The position of the z_+ wall is represented by the dashed line.

Comparison between experimental and theoretical plume flow structures

We extend here the comparison between our model and the experiments by comparing the flow obtained through PIV to the flow created in the channel by the hydrodynamic images. We plot in Fig. S10 the streamlines as well as the vertical velocity before plume formation and at (i) early, (ii) intermediate and (iii) late times of plume dynamics, for both experiments (a) and model (b). The plumes are seen to generate similar flow structures in our model and experiments, showing good qualitative agreement at different stages of the system dynamics. While the streamlines observed, or those averaged over a large field depth, are less revealing at earlier times, the plumes are characterised at all times by a strong local upward velocity.

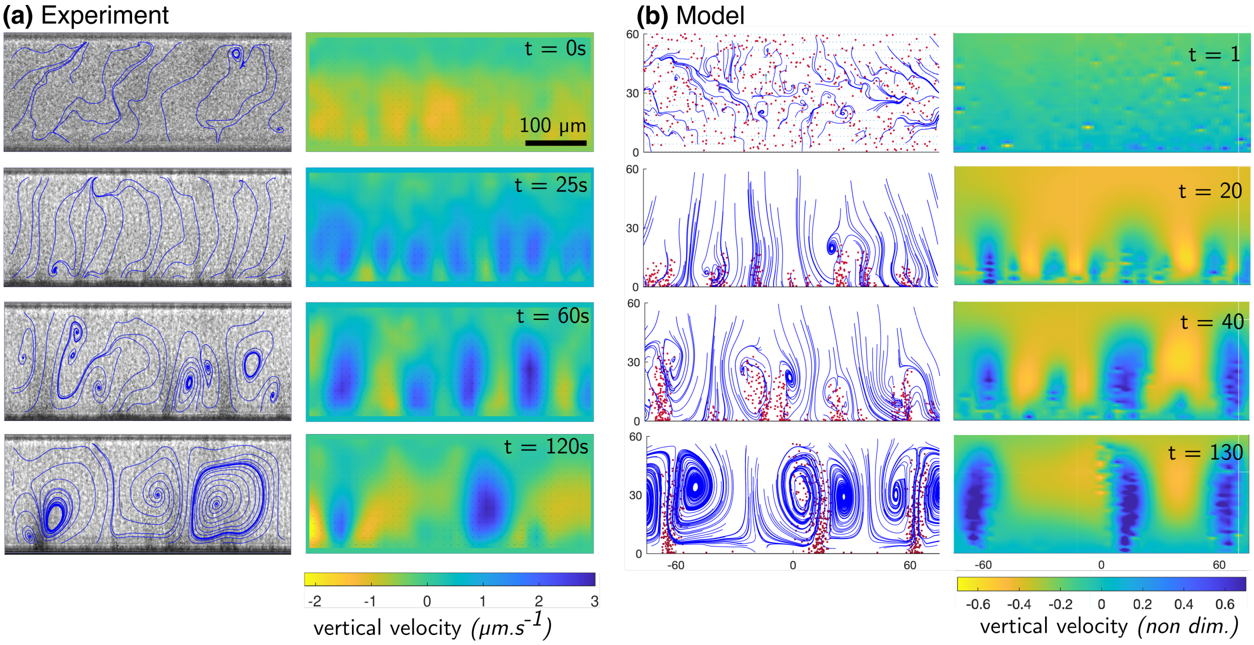

FIG. S 10: Streamlines and vertical velocity of the surrounding flow before plume formation and at early, intermediate and late time in plume formation for (a) our experiments and (b) the numerical model. The experimental panels in (a) correspond to the PIV analysis of the SM2 movie in a 200 μm channel while (b) is extracted from a simulation in a channel with $a = 60$ and $N = 2000$ swimmers.

MOVIES

Movie SM1: (steady-convection-cells-flow.avi) Two plumes in a steady flow state forming convection cells at the wall of a 200 μm capillary channel. The PIV of this movie is used for Fig. 3 convection cells in the main article.

Movie SM2: (plume-dynamics-200um.avi) Apparition of plumes at the channel wall and growth and merging of the plumes during 4 min 30 s in a 200 μm capillary.

Movie SM3: (plume-dynamics-300um.avi) Apparition of plumes at the channel wall and growth and merging of the plumes during 4 min 30 s in a 300 μm capillary. Snapshots of this movies are presented in Fig. 1 of the main article. The field of view is larger, with a frame rate of 10 fps

Movie SM4: (simulation-a60-N1500.avi) Apparition and time-evolution of the plumes for 1500 swimmers in a channel of dimensionless size 60 with an aspect ratio of 10 for the x-axis boundary condition

Movie SM5: (simulation-a100-N3000.avi) Apparition and time-evolution of the of the plumes in a channel of dimensionless size 60 with an aspect ratio of 10.

---

* These two authors contributed equally

† Electronic address: dalnoki@mcmaster.ca

‡ Electronic address: e.lauga@damtp.cam.ac.uk

[1] Nadkarni, R., Barkley, S. & Fradin, C. A comparison of methods to measure the magnetic moment of magnetotactic bacteria through analysis of their trajectories in external magnetic fields. PloS one **8** (2013).

[2] Le Nagard, L. et al. Misalignment between the magnetic dipole moment and the cell axis in the magnetotactic bacterium magnetospirillum magneticum amb-1. Phys. Biol. **16**, 066008 (2019).

[3] Blake, J. A note on the image system for a stokeslet in a no-slip boundary. Math. Proc. Camb. Philos. Soc. **70**, 303–310 (1971).

[4] Ainley, J., Durkin, S., Embid, R., Boindala, P. & Cortez, R. The method of images for regularized stokeslets. J. Comp. Phys **227**, 4600–4616 (2008).
